# Crossing mice deficient in eNOS with placental-specific *Igf2* knockout mice: A new model of fetal growth restriction

**DOI:** 10.1016/j.placenta.2012.09.012

**Published:** 2012-12

**Authors:** M.R. Dilworth, L.C. Kusinski, B.C. Baker, L.J. Renshall, P.N. Baker, S.L. Greenwood, M. Wareing, C.P. Sibley

**Affiliations:** a Maternal and Fetal Health Research Centre, University of Manchester, Manchester, UK; b Department of Obstetrics and Gynecology, University of Alberta, Edmonton, Canada

**Keywords:** FGR, IUGR, Placenta, Pregnancy, Igf2, Mouse

## Abstract

We tested the hypothesis that crossing two mouse models of fetal growth restriction (FGR) of differing phenotype would induce more severe FGR than either model alone. Female endothelial nitric oxide synthase knockout mice (eNOS^−/−^) were mated with placental-specific *Igf2* knockout males (P0). Resultant fetuses were no more growth restricted than those with P0 deletion alone. However, P0 deletion attenuated the reduced placental system A amino acid transporter activity previously observed in eNOS^−/−^ mice. Manipulating maternal and fetal genotypes provides a means to compare maternal and fetal regulation of fetal growth.

## 1 Introduction

Fetal growth restriction (FGR), the fetus' inability to achieve its growth potential, affects 5–10% of pregnancies and increases the risk of stillbirth and childhood morbidity and mortality [1]. A major cause of FGR is uteroplacental insufficiency [2]. Numerous genetic mouse models demonstrate FGR; we have characterized, in terms of placental structure/function, mice lacking the paternally-imprinted placental-specific *Igf2* promoter P0 (P0) [3,4], and the endothelial nitric oxide synthase knockout mouse (eNOS^−/−^) [5].

Near term, >90% of P0 fetuses fall below the 5th centile of wild-type (WT) weights, indicative of FGR [6]. P0 mice exhibit reduced placental size from embryonic day (E) 14, prior to onset of FGR [3]. This ‘delay’ in FGR may be achieved, in part, by an adaptive increase in placental system A amino acid transport [3]. P0 placentas have reduced surface area for exchange and increased barrier thickness, probably contributing to FGR [7], in common with human FGR [8].

eNOS^−/−^ mice, 10% smaller than WT near term [9], demonstrate aberrant uterine artery function and reduced placental system A transport [5], features characteristic of human FGR [10,11].

We tested the hypothesis that crossing these two disparate placental phenotypes would result in greater FGR than either model alone.

## 2 Methods

### 2.1 Animals

Experiments were performed in accordance with the UK Animals (Scientific Procedures) Act 1986. P0 mice were a kind gift from Professor W. Reik and Dr M.Constância, University of Cambridge [12]. eNOS^−/−^ mice were obtained from Jackson Laboratories (strain B6.129P2-*Nos3*^*tm1Unc*^/J).

eNOS^−/−^ female mice (8–12 weeks old) and P0 heterozygous males (8–32 weeks old) were mated; copulation plug discovery was designated E0.5 (term = E19.5). All fetuses were eNOS^+/−^ with either the presence (eNOS^+/−^ WT) or absence (eNOS^+/−^ P0) of P0. C57BL/6J mice (background strain) acted as controls. Animals were housed under a 12h light/dark cycle at 21–23 °C with food (Beekay Mouse Diet, Bantin & Kingman, Hull, UK) and water *ad libitum*. At E18.5, fetuses and placentas were rapidly harvested from pregnant dams, blotted and wet weights measured. Fetuses were genotyped for eNOS and P0 alleles using genomic DNA from fetal tail tips [4,5].

### 2.2 Unidirectional maternofetal clearance of MeAIB (^MeAIB^K_mf_) across the intact placenta

^MeAIB^*K*_mf_ across the intact placenta, a measure of system A amino acid transport, was measured at E18.5 as described previously [3,4,13].

### 2.3 Statistical analyses

Data are mean ± S.E.M or dot plots with median; *N* = number of litters. Variables were compared using either one-way ANOVA or Kruskal–Wallis test as appropriate. *P* < 0.05 was deemed statistically significant.

## 3 Results and discussion

eNOS^+/−^ P0 fetal weights (Table 1) were significantly less than C57BL/6J and eNOS^+/−^ WT. eNOS^+/−^ P0 mice, exposed to the dam's eNOS-deficient environment, failed to demonstrate any further FGR compared with P0 mice (Table 1) [3,4,6]. In contrast with eNOS^−/−^ mice [5,9], eNOS^+/−^ WT fetuses were of comparable weight to C57BL/6J controls. Since eNOS^+/−^ WT fetuses are heterozygous for eNOS, fetal eNOS expression, albeit at reduced levels [14], may facilitate maintenance of fetal weight. This notion is reinforced by data showing that eNOS^−/−^ mice fail to show postnatal catch-up growth suggesting that complete deletion of eNOS leads to comparably small mice both pre- and post-natally [14]. Previous data from ourselves suggested that the growth restriction observed in eNOS^−/−^ mice was likely due to placental insufficiency [5]. Evidence from the current study would appear to challenge our previous observations on eNOS^−/−^ mice and demonstrates that there could be a fetal contribution to the growth restriction. By crossing these strains, we have manipulated maternal and fetal genotype in such a way to allow assessment of maternal and/or fetal contributions to fetal growth. Future crosses to compare eNOS^+/−^ versus eNOS^−/−^ pups within the same litter may be helpful in further determinations of these relative contributions.

Placental weight was reduced in eNOS^+/−^ P0 vs. eNOS^+/−^ WT and C57BL/6J mice (Table 1), with no difference between C57BL/6J and eNOS^+/−^ WT. Placental weight reduction in eNOS^+/−^ P0 mice was similar to that observed previously in P0 mice [3,4]. Given that eNOS^−/−^ placentas are of comparable size to WT [5,9], this reduction in eNOS^+/−^ P0 placental size is likely due to P0 deletion alone. Fetal:placental (F:P) weight ratios, a marker of placental efficiency [15,16], did not differ between groups (Table 1). This lack of increased F:P ratio in eNOS^+/−^ P0 mice, seen previously in P0 mice [3,4], is likely due to eNOS deletion and reflects a ‘half-way house’ between the increased and decreased efficiencies of the P0 [3,4] and eNOS^−/−^ placentas [5,9] respectively.

**Fig. 1:**
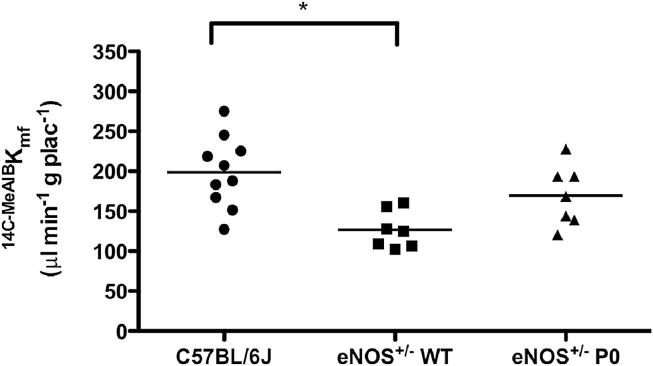
Unidirectional maternofetal transport of MeAIB (^MeAIB^K_mf_) in eNOS^+/−^ WT, eNOS^+/−^ P0 and C57BL/6J mice at E18.5. Each symbol corresponds to a mean measurement per genotype per litter. Line denotes median. *P < 0.05, Kruskal Wallis with Dunn's multiple comparison test.

At E18.5, ^MeAIB^K_mf_, per gram of placenta, was significantly lower in eNOS^+/−^ WT vs. C57BL/6J mice (Fig. 1). This difference was not observed between eNOS^+/−^ P0 and C57BL/6J nor between eNOS^+/−^ P0 and eNOS^+/−^ WT. Therefore the reduced placental system A activity in eNOS^+/−^ WT mice, previously seen in eNOS^−/−^ mice [5,9], was attenuated with P0 deletion. Interestingly, given that reduced system A transport has been observed in both eNOS^+/−^ WT and eNOS^−/−^ mice, but FGR is observed in eNOS^−/−^ only, it is possible that reduced system A transport is not necessarily a direct cause of FGR in eNOS^−/−^ mice as previously thought [5].

Our data again emphasise the importance of P0 in fetal growth and suggest that abnormal morphology and transport of P0 placentas plays an important role in the observed FGR in these mice. This model, through differing maternal and fetal genetic manipulations, has provided new data suggesting that in eNOS^−/−^ mice, fetal genotype may be a key determinant of fetal growth. Elucidating the role of maternal and fetal genetic manipulations in their contributions to fetal growth will be crucial to ensure future models of pregnancy disease are appropriate to the human situation. In the future, crossing mice with different uteroplacental structural and functional characteristics may generate models that recreate the multiple placental phenotypes of human FGR [17] and may benefit future studies both to determine disease mechanism and to test potential therapies.

## Figures and Tables

**Table 1:** Fetal weight, placental weight and fetal:placental weight ratios of C57BL/6J, eNOS^+/−^ WT and eNOS^+/−^ P0 mice. Historical observations from eNOS^−/−^ (unpublished observations from Dilworth and Kusinski) and P0 mice (taken from Ref. [4]) are shown for comparison.

	C57Bl/6J (12)	eNOS^+/−^ WT (8)	eNOS^+/−^ P0 (8)	eNOS^−/−^ (10)	P0 (16)
Fetal weight (mg)	1225 ± 15	1173 ± 38	918 ± 26^a^^b^	1064 ± 19	926 ± 16
Placental weight (mg)	84 ± 3	90 ± 1	65 ± 1^a^^b^	85 ± 6	62 ± 2
F:P ratio	14.7 ± 0.4	13.2 ± 0.4	14.4 ± 0.5	13.0 ± 0.8	15.2 ± 0.5

All data mean ± S.E.M. Number of litters used in parentheses.

a *P* < 0.001 versus C57BL/6J.

b *P* < 0.01 versus eNOS^+/−^ WT, One-way ANOVA with Tukey's multiple comparison test.
